# Subjective experiences and perceptions of learning a second language through digital games: A case study of Chinese college students

**DOI:** 10.3389/fpsyg.2022.1109370

**Published:** 2023-01-09

**Authors:** Xian Xiao, Gaoda He

**Affiliations:** ^1^School of Foreign Languages, Guangzhou Maritime University, Guangzhou, China; ^2^School of Foreign Languages, Guangdong Baiyun University, Guangzhou, China

**Keywords:** subjective experiences, perceptions, digital game, game-based learning, second language learning

## Abstract

Recent years have seen a rapid proliferation of game-based learning applied in language classes. Second language teaching and learning are being inspired by the increasingly mature game-based learning technology. Currently, educational games are regarded as a new technology with great potential. The game-based promotion of oral English has also become one of the research hotspots. This research carried out an empirical study on the subjective experiences and perceptions of using game-based second language learning to cultivate the oral English of Chinese college students. By using Unity 3D technology, “Digital Game-Virtual College” game software was developed for Chinese teenagers as the experimental carrier. Sixty-six freshmen majoring in review engineering at a university located in a first-tier city in southern China were invited as participants. Based on the courses “College English-Reading and Writing” and “College English-Listening, Viewing and Speaking,” this research explored the game-based teaching of “Words Arena Contest” and “Words Talents,” respectively. The analysis of the attitude and achievement data not only indicated the advantages and disadvantages of game-based learning, but also suggested the significant points in game-based learning software design. The results of tests and survey provide insight into the evaluation and reflection of the subjective experiences and perceptions of using digital game-based language learning. The results of this research can not only provide guidance for exploring the psychological contributors and barriers to game-based second language learning, but can also shed some light on and provide a reference for the design of game-based second language learning software.

## 1. Introduction

With the boom of information and communication technology, electronic games attached more importance in individual’s life as well as teenagers’ learning and played a crucial role in higher education. Particularly, in recent years, game-based learning aroused increasing attention, which created interactive and learner-centered teaching and learning settings (Gomes et al., 2022); hence, game-based learning came into existence. It can be said that game-based learning has become a creative teaching mode (Huizenga et al., 2009; Kapp, 2012; Schaaf, 2012; Perrotta et al., 2013; Liu et al., 2020; Hwang and Chen, 2022). Different from simply letting students play games, applying mechanics of game playing, game elements, and edutainment into curriculum design, teaching in “realistic situation” teaching can be regarded as authentic game-based teaching. In comparison with traditional teaching mode, the substantial advantage of game learning lies in the authenticity of learning resources and the situational nature of learning environment. In game-based learning, game technology can be used to provide learners in real communication situations that simulate real social activities, and can carry out real scene-oriented communication, conversation, and action interaction. In addition, participants can rely on virtual situations to obtain a variety of experiences such as story retelling, language interaction, and role-playing (Wang and Wu, 2012; Homer and Plass, 2014).

College English teaching involves the cultivation of students’ various abilities, especially their oral communication ability. These abilities include capabilities of understanding the views, emotions, attitudes, and intentions expressed by each other in social occasions, communicating on topics of common concern, choosing appropriate language forms according to the formal degree of social occasions, and appropriately expressing their viewpoints, emotions, and attitudes. In the era of rapid development of information and communication technology, the cultivation of these abilities needs the help of innovative means in teaching; hence, language learning based on digital games has become an effective way.

As Chinese college students’ mother tongue is Chinese, though they have studied English in primary and secondary schools for 12 years (9 years in some rural areas), there is a large gap compared with native speakers, especially in spoken English. Through investigations and interviews, we find that the practical problems affecting the cultivation of Chinese college students’ oral communication ability are: (1) the orientation of “teaching to the test” in middle school and the conditions of school English curriculum limit students’ opportunities to practice oral English; (2) Students’ reading ability is better than their speaking ability. Whether they can read or speak has become a stumbling block to improve their oral communication ability. (3) Students’ poor listening and speaking makes them feel ashamed to speak English in and out of class. If we can carry out language learning with the help of game-based learning and focus on cultivating oral communication ability, it will be of great value.

In this study, we focus on the subjective experiences and perceptions of using game-based second language learning, and take the cultivation of Chinese college students’ oral communication ability as a case for study.

## 2. Literature review

### 2.1. Game-based learning

Definitions of game-based learning mostly emphasize that it is a type of game play with defined learning outcomes (Shaffer et al., 2005). Usually, it is assumed that the game is a digital game, but this is not always the case (Plass et al., 2015). Game-based learning refers to the learning environment that integrates learning knowledge and skills into games, allowing learners to achieve learning through problem-solving and competition challenges while playing games (Hwang and Chen, 2022). At present, game-based learning is widely used in education. There are two schools in the research field of game-based learning.

On the one hand, there is a school study from the traction of concept, and promotes technological innovation. Since the 1990s, the concept of educational games has emerged in the world. Entertainment (as by games, films, or shows) is designed to be educational. This concept provides a theoretical foundation for the integration of education and games, and its importance has attracted more and more attention (Wang and Wu, 2012). As a teaching method, edutainment is considered to attract learners’ attention more effectively, make learners’ experience of things more lasting and remember more firmly, and is considered to be used to assist teaching (Nalan, 2015). Ghazal and Singh thought that game-based learning was one of the ways in which learner-centered pedagogy can be implemented in the classroom in order to engage and motivate learners (Ghazal and Singh, 2016). Pásztor et al. conducted a study on online assessment and game-based development of inductive reasoning. The results showed that the online training program could also be considered a pioneering enterprise in applying the advantages of the content-based method and digital game-based learning into Klauer’s training concept (Pásztor et al., 2022). Ninaus et al. assessed the fraction knowledge by a digital game (Ninaus et al., 2017). White and McCoy proposed an action research study exploring game-based learning as fifth-grade mathematics students completed a brief unit on ordered pairs utilizing game-based lessons (White and McCoy, 2019). Oksana and Elena summarized the definition and characteristics of the concept of edutainment, its potential in modern educational technology, and its methodological value as a whole (Oksana and Elena, 2015). In particular, the application of edutainment means in teaching is also referred. Kiili, Moeller, and Ninaus conducted a study, which indicated that the game-based training group improved their conceptual rational number knowledge significantly more strongly than the control group (Kiili et al., 2018). In particular, improvement of the game-based training group was driven by significant performance increases in number magnitude estimation and ordering tasks. In recent years, based on edutainment, more new concepts have emerged in the field of game-based learning. Scholars try to accelerate the development of game-based technology through these new educational concepts. Such as game-based learning, gaming, educational games, digital creative play, e-learning, and so on (Tomczyk and Walker, 2021).

On the other hand, with the help of the new achievements of information and communication technology, carry out technology integration and application. For example, Versailles 1,685: a game of intrigue, jointly produced by the French game company named Cryo and the multimedia division of French broadcasting company Canal +, is mainly an adventure game for court conspiracy. There are also some widely used games, such as Romance of Three, SimCity, Uncharted Waters Online, The Sims, Hospital Sim Pro, etc. Game developers popularize these professional game software to publicity mainly catering to people’s entertainment needs. Li et al. presented a novel method on measuring ease of use of mobile applications in e-commerce retailing from the perspective of consumer online shopping behavior patterns (Li et al., 2020). However, these game software are gradually applied to the field of education and become digital learning games. Through the use of game software, Alt and Raichel designed an experiment and examined the potential of employing a gamified problem-based learning to increase digital literacy skills and creative self-concept among undergraduate students (Alt and Raichel, 2020). Sun et al. explored the relationship between collaborative problem-solving behaviors and solution outcomes in a game-based learning environment (Sun et al., 2022). In recent years, the latest achievements of brain science have also been applied to the teaching design and research of discipline study. The theories related to cognition, anxiety, motivation, memory, reward, and punishment in the field of pedagogy can also provide theoretical basis and design ideas for game-based learning (Mccandliss, 2010). These studies bridged the gap between the cognitive neuroscience and educational science.

### 2.2. Learning a second language through digital games

Generally speaking, mobile-assisted language learning is a subarea of mobile learning in which integration of new mobile technologies into teaching and language learning has been a primary focus (Osifo, 2019). In maths accumulated in remote learning, an interesting and valuable study was performed, and an effective computer-based intervention program for students was designed by several Hungarian scientists. The findings indicate that both the mathematical skills and the subject knowledge expected of primary school students can be significantly and effectively developed in a computer-based personalized environment (Réka and Gyöngyvér, 2022). Teng achieved a high value study that aims at filling the theoretical and practical gaps by examining the impact of watching a documentary TV program on incidental vocabulary learning and assessing to what extent this learning is influenced by learner-related factors (Teng, 2022). These findings have pedagogical implications for using computer-based teaching resources, especially digital games to enhance second language learning.

On the whole, game-based learning is used more in K12 stage. For instance, in the process of learning chemical elements and equations, design breakthrough and treasure hunting games, use heroic narration game designing in history subject, design letter games in English learning, moreover, design number sense games for children with dyscalculia. In recent years, China has also established a professional committee on game-based education, which mainly focuses on game-based teaching workshops case teaching, platform construction, and product research and development in primary and secondary schools. Education sustainability has continued to evolve, which provides greater opportunities for interaction between colleges and their surroundings (Ghani et al., 2022).

Nevertheless, at present, in the field of higher education, little attention has been paid to game-based teaching, and researches on game-based learning in the field of sustainability in higher education are rare, too. In language learning, particularly, in college students’ second language learning, there is only a minor amount of research attached to virtual reality immersion learning. What is more severe is that in oral communication teaching for higher education, researches on the theory and practice of game-based learning are insignificant. The rationality, applicability, and effectiveness of using game learning require exploring.

### 2.3. Rationale and research questions

The subjective experiences and perceptions mean that learners, based on existing experience, through the interaction between individuals and the environment, simulate various tasks and activities to gain new knowledge, reconstruct the knowledge system, and reflect on the experience obtained, so as to achieve the goal of internalizing knowledge. Motivation is the psychological tendency or internal drive to stimulate and maintain the action of the organism and guide the action to a certain goal. Motivation is closely related to the generation of behavior. The occurrence and maintenance of learning behaviors need certain motivational strategies. The use of learning games can make students feel the joy of games while learning, which can well stimulate students’ learning motivation and maintain their input on learning behavior.

Game-based learning is supported by the mobile terminal. The teaching situation created by the educational game avoids feeling bored in the traditional classroom teaching situation to meet the students’ personalized learning. Through a series of teaching activities, students’ learning behavior input is maintained, so that learners can acquire knowledge in the process of participating in the game situation and completing tasks, so as to achieve the teaching goal. Learning behavior input reveals the extent to which learners constantly interact with information to drive their development in the process of interaction with the learning environment. According to the research of McLoughlin and Lee, digital communication tools and ubiquitous networked applications have had a profound effect on behavior, particularly those of young people whose medium and metier it is (McLoughlin and Lee, 2010). Games can be used to motivate and engage users in using regular system and, in the end, support learners in achieving better learning outcomes (Schobel et al., 2021). McLaren et al. summarized the lessons from a study with a digital learning game, and uncovered some interesting differences between students working in school versus at home, as well as within and without hints (McLaren et al., 2021).

The core of game-based learning lies in the game-based teaching environment, which includes three characteristic elements: first, learners who have strong desire to study, teachers with information-based teaching quality, and effective virtual reality resources suitable for classroom teaching. Teachers guide students to experience educational games with the support of technical environment; Secondly, with the help of mobile handheld devices such as smartphones and iPads, digital games are used as teaching and cognitive tools; the third is the deep integration of educational game function and teaching content. Learners acquire knowledge in the process of participating in the game situation and completing tasks. Specifically, the application of learning games is embedded into the general process of teaching activities through different forms of activities, and the entire environment completes the learning tasks in question-based games.

As far as Chinese college students are concerned, it is imperative to learn a second language through digital games. From the perspective of language environment, interpersonal communication between China and English-speaking countries is not frequent enough, which is also a reason for difficulties. From the perspective of individual English learning, the actual situation is that most people are rarely given occasions where they have to communicate in English from primary school to university, and have fewer opportunities to use English except for examinations. This is the so-called problem of lacking a sufficient language environment. As a second language, English learners are naturally less familiar with the language expression and grammar usage than their mother tongue, which they have mastered since childhood. It requires a lot of practice to learn to express as skillfully as native speakers, but most people cannot. Therefore, how to use digital games to cultivate the oral English of Chinese college students is a very urgent task.

This study mainly focused on subjective experiences and perceptions of learning a second language through digital games. A case study of Chinese college students to cultivate oral English was used in the study to support the research topic about how digital games can help Chinese college students learn languages. This is currently the cutting edge of language learning. For example, some scholars are investigating school teachers’ use of technology in teaching Chinese as a foreign language. This study aimed at addressing the following research questions:

1. How do people demonstrate the role of digital games on subjective experiences and perceptions of learning a second language through digital games?
2. How do people demonstrate the effect of digital games in the cultivation of oral English of Chinese college students?
3. How do people test the language awareness, cognitive awareness, communicative awareness, and emotional awareness through digital games?

## 3. Research design

### 3.1. Game software

This study intends to explore game-based language learning, with the help of the “Digital Game-Virtual College.” “Digital Game-Virtual College” is a word reciting software aided with pictures developed for English learning. It is essentially a game software, which can be used for students’ oral English practice. Therefore, it can also be realized as a video system with the teaching function. In this study, Unity 3D engine is applied to design digital game software. The details of some modules of the engine are as follows:

1. Game Object: All objects in unity3D, such as characters and various geometries, are collectively referred to as objects, and each object can be controlled by adding components and attaching scripts.
2. Component: A component is a tool added to an object to realize different functions. For example, Transform can set the position, rotation, and scale of the object; Collider can control the collision effect of objects.
3. Physics Engine: Unity 3D has a very comprehensive physics engine, which can simulate the physical characteristics of objects in real life, including rigid bodies, colliders, triggers, joints, rigid body forces, etc., greatly reducing the difficulty of game development.
4. Camera: Camera is one of the most important game objects in Unity 3D, capturing and displaying game scenes for players. There can be many cameras in a scene, which can be rendered and displayed in a manually given order, and can also be adjusted to render and display in a certain area of the display.
5. Renderer: Rendering is a component of an object. Like 3D modeling, it combines models, materials, maps, animation, light and shadow effects, and other special effects in the scene to change the image or mesh drawing method of particles to achieve high-quality display effects.
6. UGUI (graphical user interface): UGUI is the UI system officially launched by Unity 3D. Common controls include Canvas, Text, Image, Button, etc. It can also realize human-computer interaction through external devices such as mouse and keyboard.
7. Assets: It is an important module of engineering resource management. All the original resources used in the project, such as resources, codes, configurations, and libraries, can only be recognized and processed by Unity 3D when placed in this folder. It can also import materials made by other software, which reflects the convenience and practicality of Unity 3D.
8. Script: Scripts can be attached to objects to implement various game logic, such as processing user input, manipulating game objects, detecting physical collisions, generating new game objects, setting light, etc. Unity 3D exposes a well-documented API that makes these operations easy.
9. Animation: The animation component can edit the physical animation, set off the specified object, and achieve complex animation or interaction effects.
10. Particle System: The particle system creates the effect of disordered particles by repeatedly painting several materials. It is often used to achieve atomization effects, including tail flame, air jet, and other special effects. It is mainly composed of three parts: emitter, renderer, and animation. You can change the Inspector attribute panel to adjust the emission density, emission direction, and other attributes of particles.

Unity 3D has a very simple visual editing interface. Researchers can quickly operate and create games. The editing interface of Unity 3D mainly includes menu bar, toolbar, Project, Hierarchy, Inspector, Sense, Game, etc.

1. Project (drawing view): includes all available resources of the whole project, such as models, scripts, etc.
2. Hierarchy (hierarchical view): the game object that stores the entire project, used to show the parent–child relationship between various objects.
3. Inspector: displays parameters such as components and properties of game objects.
4. Scene (scene view): You can drag the game object into the Scene scene, adjust the position and angle of the object, and set detailed parameters in the object’s Inspector interface.
5. Game view: This view is part of the scene rendered by the camera, which is also the picture that players can see when playing games.

**Figure 1:**
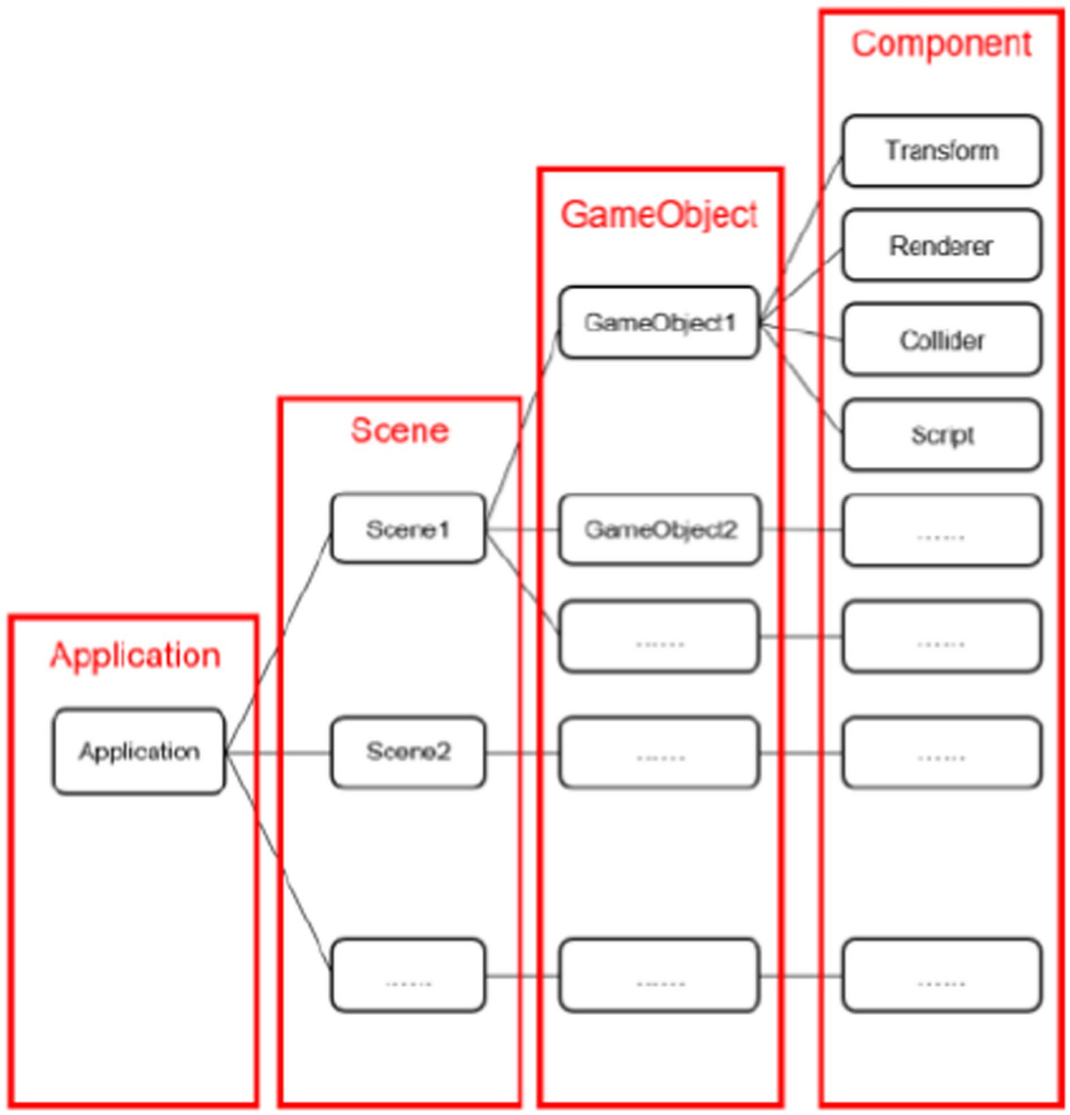
Development process of the game software.

As is shown in Figure 1, the Unity 3D development process can be mainly divided into four parts: Application, Scene, GameObject, and Component.

An Application is a fully functional game encapsulated by Unity 3D packaging and output. It consists of multiple separately constructed Scenes. In each scene, multiple objects can be set, including game objects, trees, ground, sky, UI display, etc. Each object can be configured with multiple components to achieve the effects of moving, rotating, colliding, and generating thrust and gravity.

The scripts in Unity 3D are different from traditional programs. The code of traditional programs will run continuously in the loop, while Unity 3D runs the script intermittently by calling some functions declared in the script. These built-in declaration functions will respond in order after Unity 3D is activated, so they are called event functions.

MonoBehavior is the base class in Unity 3D. All created scripts can only be directly run in Unity 3D if they inherit from the MonoBehavior class. If the script is written in C #, you need to write your own class inheritance to explicitly inherit MonoBehavior. If the script is written in JavaScript, it will automatically inherit MonoBehavior implicitly. This article uses C # language to write the script.

**Figure 2:**
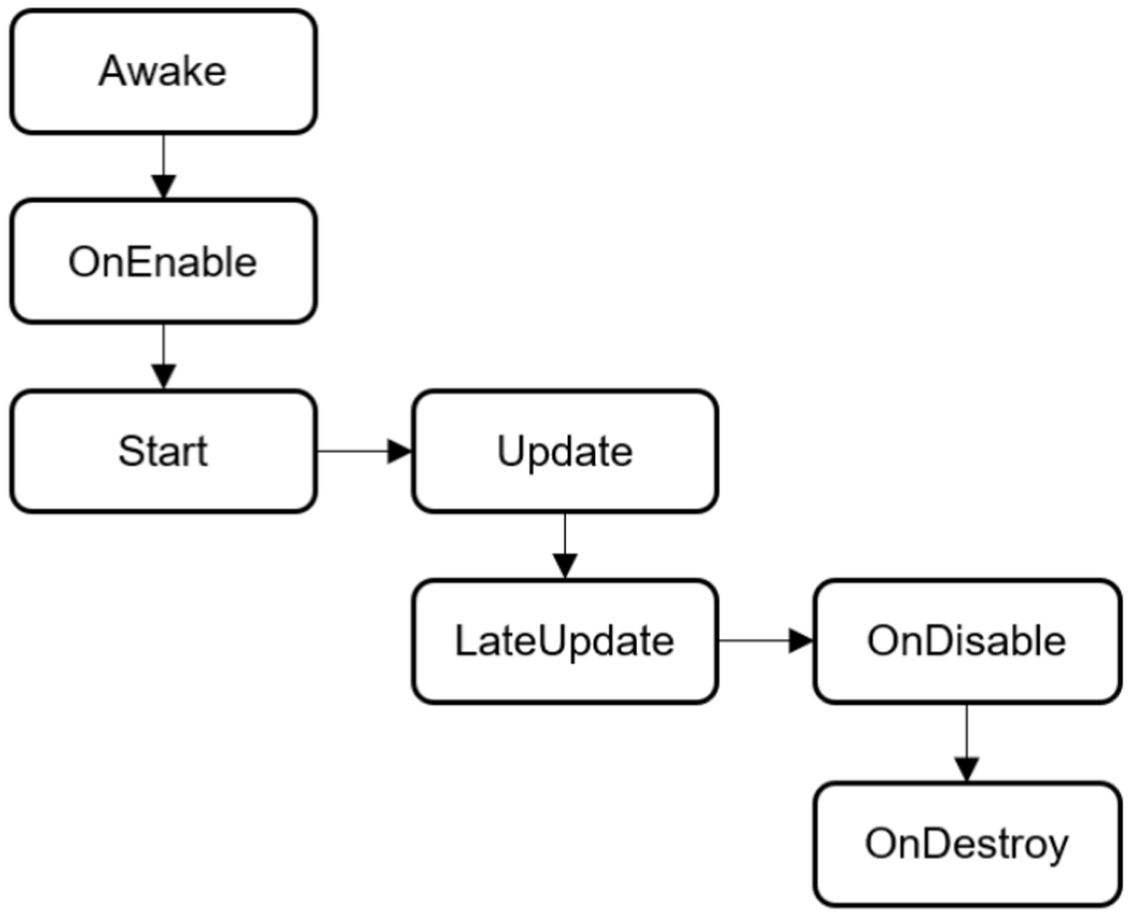
Execution sequence of common event functions of unity 3D.

Event functions are common methods in the MonoBehavior class. When creating a new empty script in Unity 3D, the script will have two event functions, Start() and Update(), by default. In addition, event functions include Awake(), OnEnable(), LateUpdate(), OnDisable(), and OnDestroy(), which reflect the life cycle of the script running process in terms of time and logic. The general running sequence of these event functions is shown in Figure 2.

As far as the realization of human-computer interaction function is concerned, the game-based learning software designed in this paper adopts GUI (Graphical User Interface), which is a graphical display of computer operation, more convenient than the command line interaction mode used by early computers, and an important medium in the human-computer interaction process. Through GUI, the internal data of the computer program are presented to the user in a visual form, which makes the command operation more humanized.

UGUI is an officially launched new UI integration system, which is flexible, fast, and visual. It can quickly establish UI interface without relying on programming. Common controls include Canvas, Text, Image, Button, etc. Common controls are described below.

1. Canvas: When creating a new GUI, the system will automatically generate a Canvas, and all GUI elements will be displayed on it, which will be automatically set as parent–child relationship. You can drag the mouse to adjust the position. There are three rendering modes:
  - Screen Space: Overlay: render the UI elements on the screen at the top of the scene, and the size of Canvas will change with the screen resolution;
  - Screen Space: Camera: Canvas will be in front of the specified camera, and different cameras will have different display effects;
  - World Space: Like game objects, Canvas can adjust its position and posture through RectTransform, and can also move with other objects in the scene.
2. Text: Text control is used when GUI needs to output text information. You can customize the font, size, format, and other attributes in Text.
3. Button: Button controls are generally used to handle human-computer interaction events. You can click the mouse and press the keyboard to interact. Keyboard and mouse control computer is very important in game operation. There are many keys on the computer keyboard. In 3D games, W, S, A, D, Q, and E are often used to move forward, backward, left, right, up, and down. Frequently used mouse operations include left and right mouse button clicking and long pressing, mouse movement, mouse wheel scrolling, etc., which are used to rotate, zoom in, and zoom out the camera perspective. In the game engine of this learning software, read three common functions of key operation: Input GetKeyDown(), Input. GetKeyUp(), and Input. GetKey(), corresponding to the press, lift, and long press of the key.

**Figure 3:**
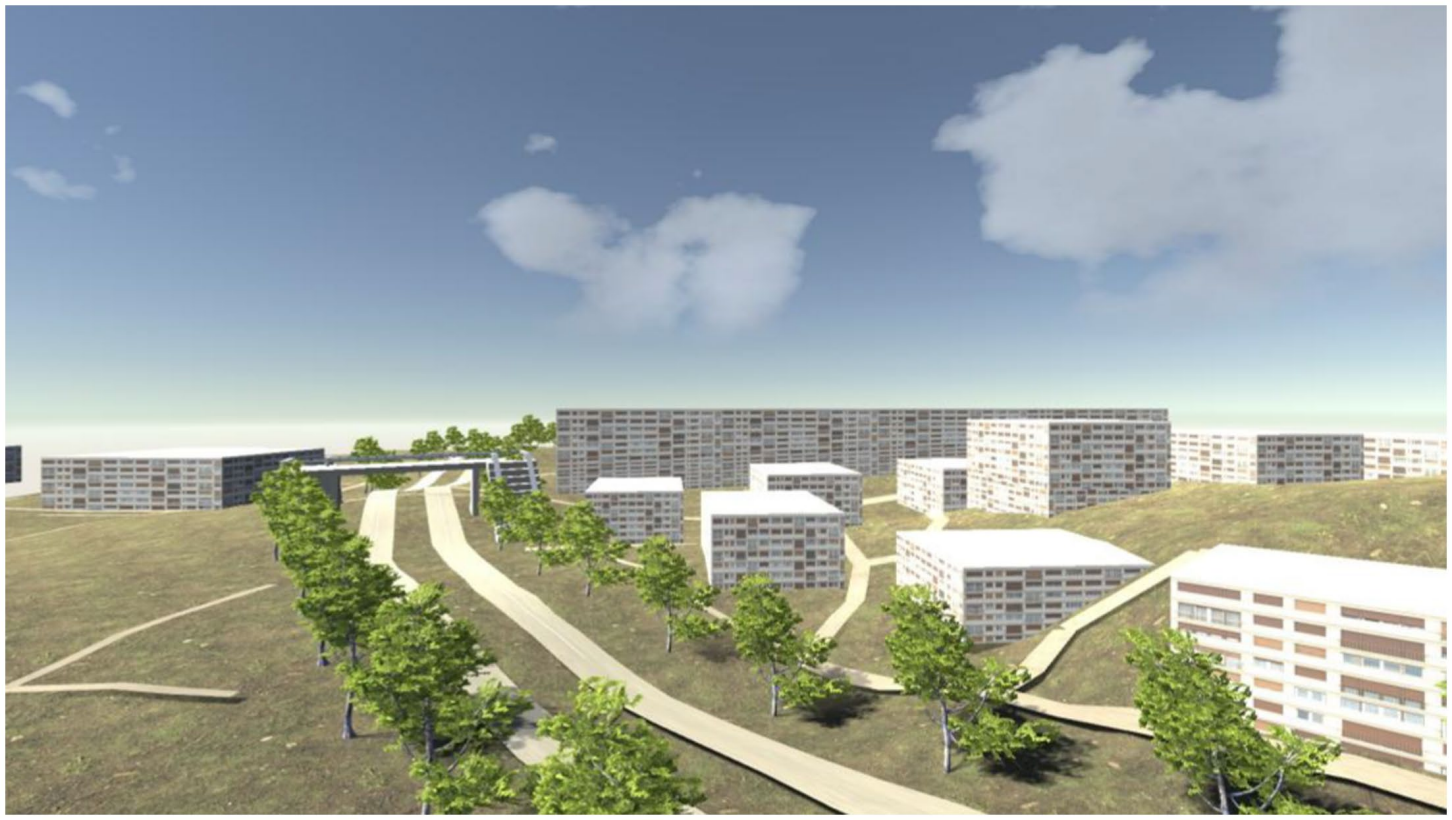
Game software “Digital Game-Virtual College.”

“Digital Game-Virtual College” software provides interesting pictures and example sentences for each word. A word corresponds to a picture, and the graph is used to establish the relationship between the word and the context, so as to deepen the player’s memory of words. One can also interpret words and test vocabulary through interesting contents provided by word TV and word broadcasting. Using the game embedded in “Digital Game-Virtual College,” we can also carry out English sitcom practice of role-playing. The game interface of “Digital Game-Virtual College” is shown in Figure 3.

### 3.2. Participants

The participants in this study are 66 freshmen majoring in engineering in universities in a first-tier city in southern China. Their mother tongue is Chinese while taking English as their first foreign language. These students graduated from middle schools in cities and counties of more than 10 provinces in China. According to the survey, the majority of them have never really taken English audio-visual and oral classes before entering the University. Oral communication is limited to answering teachers’ questions in class. The listening materials are basically the recording of the text and the simulation testing questions for the college entrance examination, and there is a lack of listening materials in the real English context. Among the above participants, male and female students accounted for 77.27 and 22.73% respectively, aged 17–20 years, of which 59.09% were from villages and towns and 40.91% were from cities.

### 3.3. Experiment design

This study is conducted in the courses of “College English-Reading and Writing Course” and “College English—Listening, Viewing and Speaking Course.” Before the beginning of the course, half of the participants, 33 students, participated in the oral training of “Digital Game-Virtual College” for half an hour per day. The content of the game is “Words Arena Contest;” the other half of the participants acted as the control group, not game learning, but traditional English learning. “Words Arena Contest” game, every 14 days for a competition season, a month is divided into two seasons, including qualifying and practice, and the results of a season are rewarded according to the ranking. By adding friends, you can see friends’ learning trends and sprint the ranking lists together. Topics of vocabulary include daily oral English, slang, self-introduction, hobbies, food and restaurants, travel, and other subcategories.

After completing the task of learning the whole “Words Arena Contest” game, one can attend the game of “Words Talents.” Similarly, 66 students in the whole class were divided into two groups for comparison. The 33 students in the experimental group, holding personal smartphones, spend 30 min a day repeatedly following the game of “Words Talents” with an English conversation in advance to practice oral English. Through the “Words Talents,” students complete the learning plan every day and stick to it for 4 weeks. Those who succeed in the challenge can be rewarded. After completing the first stage (the first 4 weeks) of game learning with social networking as the main content, carry out the second stage (the last 4 weeks) of game training to further upgrade the game content and turn to the cultivation of emotional expression ability. Vocabulary topics include work, shopping, movies and TV dramas, leisure and entertainment, and other subcategories.

In the game learning practice, every week is a cycle. The tasks of each week mainly include (1) half an hour of “Words Arena Contest” on the game platform; (2) Use the functions of “Words Arena Contest” and “Words Talents” in the word game learning software “Digital Game-Virtual College” to acquire vocabulary; (3) Fill in the reflective logs of practice experience; and (4) Classroom teaching experiments. In class, the teacher gives a conversation scene which is consistent with the game content, and invites the participants to express and communicate orally.

Three phases of experiments were conducted in this study. The experimental process of each phase is shown in Table 1. Each issue includes five links: setting rewards, clarifying objectives, scenario construction, learning tasks, evaluation, and summing-up.

**Table 1:** The experimental design of game-based learning in “Digital Game-Virtual College” application.

Game types	Implemented courses	Contents for learning	Main modes of games	Learning objectives
“Words Arena Contest”	College English-Reading and Writing	Daily conversation, slang, and idioms learning experience exchange, self-introduction, introduction to food in restaurants, conversation in traveling, introduction to interests and hobbies	Qualifying and Practice, adding friends, and cooperate with each other to sprint the ranking list	Enhance students’ linguistic awareness and cognitive awareness, including enlarge English vocabulary, improve their spoken English, be familiar with English idioms, master English grammar, and introduce themselves about personality, expertise, companions, food and scenery, etc.
“Words Talents”	College English-Listening, Viewing, and Speaking	Role-play activities: the boss’ work arrangement and tasks handover as a new employee, play the transaction between the seller and the buyer in the market, role play as actors and actresses in TV series, role-play as friends, how to cure his (her) depression and make him (her) happy	Role-play, Participate in scene-based games	Enhance students’ communication awareness and emotional awareness, including improve their social ability and emotional expression ability, and master the application of key situational language of their respective roles in life

In the sector of incentive setting, it is mainly to clarify the reward mechanism of learning a second language through digital games, stimulate students’ enthusiasm to participate in teaching activities, and try to make participants understand the evaluation methods of academic performance. In the stage of clarifying the objectives, the teaching tasks are deployed according to the teaching objectives and key contents. In the scenario construction stage, the designed scenario story is provided to the participants with the form of story, providing them a certain role in the story, so that the participants can enter the virtual digital game environment. In the stage of fulfilling the learning task, the researchers try to mobilize students’ enthusiasm, and carry out situational teaching based on role-playing and game software, such as the self-introduction in social networking, communication at dinner, conversation between two people during the traveling, playing main roles in movies and the TV series, giving assignment in tasks between superiors and subordinates at work, or carrying negotiation between customers and merchants, etc. In the evaluation and the summary link, at the end of the game learning, score the students according to their participation. At the same time, guide the students to think deeply about what has been obtained from the digital game in their learning process, try to find out the deficiencies and aspects which need a further improvement in the process.

### 3.4. Data collection and analysis

After the fulfillment of all the experiments, we conducted empirical research in two aspects: one is the test and the other is the questionnaire survey. Accordingly, data were collected during these experiments. Through these empirical studies, this paper verifies the effect of learning based on “Digital Game-Virtual College” game in cultivating Chinese college students’ oral English. The test results are shown in Table 2. The results of the questionnaire survey vary from four aspects: language awareness, cognitive awareness, communicative awareness, and emotional awareness, which are shown in Table A1. The results of the questionnaire survey before and after their second language learning through digital games are shown in Table A2. In order to better guide the design of game-based learning software, this study also conducted a questionnaire survey on the evaluation of game-based learning software design. The results are shown in Table A3.

**Table 2:** Test results of using “Digital Game-Virtual College” as game-based learning software.

Contents	Time	Control group		Experimental group		Mean difference (MD)
		Mean score (M)	Standard Deviation (SD)	Mean score (M)	Standard Deviation (SD)	
Vocabulary	After the first Season	67.04	11.92	75.82	9.67	8.78
	After the second Season	72.25	10.13	80.36	8.54	8.11
	After the third Season	76.36	12.63	85.78	10.32	9.42
	After the fourth Season	78.93	9.72	89.12	8.76	10.19
Oral expression ability	After the first Season	64.12	10.65	71.03	9.48	6.91
	After the second Season	65.74	8.62	75.32	7.91	9.58
	After the third Season	67.43	9.13	79.87	8.92	12.44
	After the fourth Season	70.35	10.59	85.41	9.45	15.06
Social interaction	After the first Season	63.57	11.76	72.34	10.83	8.77
	After the second Season	66.79	10.52	77.91	9.57	11.12
Emotional expression	After the first Season	62.95	9.48	68.53	8.05	5.58
	After the second Season	64.82	8.64	74.27	8.56	9.45

Results revealed that, 33 students in the experimental group used “Digital Game-Virtual College” application to carry out game-based learning, compared with the 33 students in the control group, their performance on vocabulary, oral expression, and the emotional expression ability was better. In terms of vocabulary, the game-based training students scored more than 8 points higher than the control group in every season, specifically, after the fourth season, the average score was more than 10 points. As far as the performance of oral English expression is concerned, the students in the experimental group are more outstanding. Results in Table 2 suggested that, as the game-based training proceeds, the average score between the experimental group and the control group is becoming increasingly differentiated. The difference has gradually increased from 6.91 points after the first season to 15.06 points after the fourth season. After the first stage, in terms of socializing ability and emotional expression ability, the average score difference was 8.77 and 5.58, respectively; however, after the second stage, the average score difference increased to 11.12 and 9.45. These results reveal that the use of “Digital Game-Virtual College” application for game-based learning is effective against the cultivation of oral communication skills of Chinese college students, especially the ability of oral English expressions; students have been greatly improved after learning through game. Their emotional expression ability was also improved. In terms of vocabulary, with the improvement of students’ proficiency in using the game software, their performance can also be significantly improved.

The results of the questionnaire indicate that, in terms of language awareness, cognitive awareness, communicative awareness, and emotional awareness, after learning through games, the students in the experimental group generally felt more confident and improved as compared to the control group. From Table A1, it revealed that, among them, the biggest improvement is reflected in emotional awareness. For instance, after game-based training, students in the experimental group were more willing to act as protagonists in movies and the TV series and act out love stories. Among the 33 students, only 4 students chose “uncertain,” and 6 students chose “not quite certain;” the number of students choosing “certain” and “quite certain” reached 26 and 13, respectively. Among the 33 students in the control group, 10 and 15 chose “uncertain” or “not very certain;” only 15 and 5 chose “certain” and “quite certain.” Moreover, in terms of communication awareness, in particular, the cultivation of the ability of negotiation between customers and merchants in shopping, the students in the experimental group are more certain that they can actively try to be sellers and pursue maximum profit. There are also notable differences in the cultivation of awareness in other aspects.

It is not hard to see from Table A2 that after the game-based learning, students generally prefer to use video games to help their learning, and the proportion of preference has increased from 74.24 to 93.94%. The findings indicate that game-based learning has become a more established learning mode. 98.48% of the students are quite agreeable to accepting this teaching method; they believe that game-based teaching can promote the establishment of harmonious relation between teachers and students; they found it challenging and fulfilling to design the learning contents as a game for peers to use. 95.45% of students hold that in game-based learning, they not only exercised their practical ability and oral expression, but also cultivate their spirit of teamwork. 93.94% of students believe that confronting the “information natives,” game-based learning is one of the main ways toward future education; compared with other teaching methods, it is easier to acquire knowledge in game-based teaching, and it is also easier to arouse self-reflection. The students have expressed their willingness to accept game-based learning in future. 92.42% of students believe that, compared with traditional teaching, assessment and behavioral performance in game-based learning are more reasonable and scientific.

However, the results of the questionnaire also reveal that, compared with traditional teaching, the changes and quality improvements brought by game-based learning to students are not immediately apparent. In terms of the consolidation of theoretical knowledge or the improvement of professional skills, the transformation of learning attitudes or the flexible application of knowledge and skills in work, or even the enthusiasm in class, a lot of students have not achieved perfectly. A lot of students have not achieved perfectly. A similar situation exists in terms of stage presence and performance, verbal dexterity, practical ability, leadership, and self-confidence. A considerable number of students harbor reservations about this game-based learning experiment. For example, among the 66 students, respectively, 8, 14, 11, and 9 of them thought that the experiment was too focused on the game process, and the process was too relaxed, the experience was not strong or even poor, and that they learned in the classroom was not so applicable in practice. Therefore, results of the study suggest that it is impossible to carry out game-based learning overnight, game-based teaching is a marathon rather than a sprint, and it takes time to carry out more effective learning.

In an attempt to have a better analysis on what issues should be paid attention to in designing and developing game-based learning software, we conducted a questionnaire aimed at evaluating the software designing, and the results are shown in Table A3. Concerning about the factors of software design, the results revealed that 61 of the 66 students think that “Educative nature” is very important or important; 60 of them think “Entertaining nature,” “Participation,” and “Experience design” is very important or important. According to the results, 43 students attached the “Educative nature” element to the greatest importance, while the “Teamwork” element ranked second (37 students), followed by elements as “Entertaining” and “Experience design” (35 students for each). To the less extent is “Participation” element (34 students), while the two least important elements are “Artistry” and “Rewarding;” take only 22 and 19, respectively. Thus, it can be seen that, as college students, they can treat the new teaching mode rationally, and regard the instructive nature of game software as the most important factor. In addition, students are generally in favor of a game software with teamwork, entertaining nature, participation, and participation. It is not difficult to imagine that if the game software put into the market does not perform well in the “game” function, it will be difficult to gain users’ appreciation. As for the natures of “Artistry” and “Rewarding” of game software, because it is mainly aimed at the cultivation of oral communication skills of Chinese college students and belongs to the design of learning software, these two design indicators are not so important, so they are not the most concerned. According to Table A3, students are full of expectations for both knowledge-embedded games and instructive games, especially in terms of interactivity, fun, and game play. Regarding the issue of how to consider the reward mechanism in the design of game-based learning software, more students think that the external means reward mechanism and the virtual reward mechanism are more important.

## 4. Evaluation and reflection

### 4.1. Motivations of game-based learning

In our survey, only 4 of the 66 college students chose “dislike” video games, which shows that there is no significant difference between adult college students and children in their interest in video games. 68.18% of the subjects believed that they would not reduce their original learning time by playing video games, which shows that adults have a certain degree of self-controlling over video games; hence, there is a reasonable feasibility and basis for implementing game-based teaching. Taking advantage of game-based learning software can promote students’ cognition on the courses, improve the participation rate, and enhance their learning motivation. In this study, we found that the game-based learning experiment provided an experimental teaching scene for freshmen majoring in engineering in universities in a first-tier city in southern China. Under the teacher’s organization, students can make full use of this game-based learning software to carry out their learning of various contents.

Therefore, it is believed that game-based learning can mobilize students’ enthusiasm in oral English learning. Game-based teaching might not enter the real or virtual game, but to apply the principles and mechanisms of the game so as to make the learning process more pleasant while participating in the game. The contents to be learned and the contextual stories are naturally integrated together. The game interface and game scenes can also meet learners’ aesthetics and bring learners a happy emotional experience. This kind of oral English teaching based on game learning software essentially reflects people’s interests in new means, the pursuit of a sense of participation, the need for beauty, and the willingness to be free and relax. Therefore, as long as the game learning software which is suitable for the cultivation of college students’ oral communication ability is developed, it will be definite of high application value practically.

### 4.2. Project group form of game-based learning

To carry out game-based learning and cultivate college students’ oral communication ability, we also need to arouse students’ awareness of social cooperation. In other words, teachers and students both need to jointly design game-based learning projects according to students’ requirements, emphasize interactive learning, and create more opportunities for students to learn from each other by using situational language teaching experiments. In this way, we can further demonstrate the formation of game-based learning project group, and carry out more experiments in teaching cooperatively among members, so as to provide more opportunities in realistic learning for students. This real cooperative game-based learning will increase the synchronous discussion. On the one hand, it will enable the students in the same group to improve the learning effect through real learning tasks and shared learning objectives, which has a positive impact on eliminating the loneliness of individual learning; On the other hand, it can also enhance the sense of social existence between different project groups, promote players to form good interpersonal relationships in the learning process, and establish a sense of belonging to the learning community.

### 4.3. Integration of form and contents on game-based learning

Digital game learning is not a traditional form of learning, nor is it a game. The design of game-based learning includes both formal design, focusing on game elements, and contents design, focusing on cognitive teaching. The contents of these two parts have different emphases. The cognitive content focuses on knowledge, including the core cognitive content and its ancillary content, which focuses on the cultivation of students’ oral communication ability. The design of game elements focuses on entertainment, including the interactive design of game situation and plot, and the design of reward and punishment mechanism, sound effect, and animation effect. It focuses on oral teaching means. To improve the cultivation of Chinese college students’ oral communication ability based on digital game learning, the essential requirement is to combine games with foreign language teaching to achieve the purpose of learning and mastering oral knowledge in games.

In reality, there are some problems existing in the design of digital game learning at home and abroad presently, such as the teaching contents and games are not so well combined. Many products are teaching software with some functions of game play, and there are many contents irrelevant to learning in the game, which will divert students’ attention and waste teacher’s time, affecting the learning effect. From the perspective of the form of expression, some contents seem stiff. Although they may be more in line with the tastes of schools and teachers, from the perspective of educational philosophy, they cannot reflect the goal of learning. The main difficulty in the application of digital games to the cultivation of Chinese college students’ oral communication ability is how to realize the unity of education and game. If the game is less playful and the game elements are single, the oral English teaching process will be less interesting. If the game is less educational and the cognitive content is simple, it will lose its educational significance. The cognitive content and game elements of game learning need to achieve a certain balance, which cannot be neglected. Excessive emphasis on cognitive content or entertainment will have an impact on the learning effect.

Second language learning game design is essentially an educational game. It must conform to some characteristics of educational games: it should have clear teaching objectives and feedback mechanisms, which can attract students’ attention. Meanwhile, the software needs to be simple and easy to operate with clear instructions. The reward mechanism of game design needs to enhance the confidence of oral English learning, reduce the anxiety of oral English communication, and promote the learning reflection and knowledge system construction of teachers and students. This research focuses on the design of game learning software, and carries out research and analysis in the field of education sustainability. The relevant results are worthy of reference for game learning software designers.

## 5. Conclusions and future work

Language learning games aim at promoting language learning through digital games, and the coordination of entertainment and learning in the game itself is the key to making game-based language learning both instructive and entertaining. For addressing the issue of cultivating Chinese college students’ oral communication ability, this research used the game software “Digital Game-Virtual College,” taking the courses of “College English—Reading and Writing” and “College English—Listening, Viewing and Speaking” as the case study among 66 freshmen who are from an engineering-oriented university in southern China. We conduct case practice and theoretical analysis on game-based second language learning from the perspective of subjective experiences and perceptions. As far as the comparative experiment between the control group and the experimental group is concerned, the four aspects of vocabulary, oral expression ability, social ability, emotional expression, and four aspects of language awareness, cognitive awareness, communicative awareness, and emotional awareness are tested. The survey fully demonstrated the effect of the “Digital Game-Virtual College” game software in the cultivation of college students’ oral communication ability. Judging from the test results, the students in the experimental group had better performance than the students in the control group in terms of vocabulary, oral expression, social ability, and emotional expression ability after game-based learning. The results of the questionnaire indicate that the students in the experimental group were more certain to improve in four aspects: language awareness, cognitive awareness, communicative awareness, and emotional awareness. As far as the experimental group itself is concerned, a questionnaire was conducted for the comparison before and after game-based learning and the evaluation of the software design of game-based learning. The empirical results show the advantages of game-based learning; meanwhile, it also reveals that the change and improvement of oral communication ability brought by game-based learning is not achieved overnight, and requires long-term learning. In addition, combined with the students’ recognition of the entertainment, participation, experience design and other characteristics of game-based learning software, the investigation on the game software itself also revealed the ideas and directions of how to design game-based learning software scientifically and rationally. On this basis, focusing on the motivation, form of program group, and the unification of form and content design in game-based learning, this article further evaluates and reflects on the problems on the subjective experiences and perceptions of using digital game-based second language learning for Chinese college students.

Game-based second language learning is endowed with a wide foreground, especially in the development of oral communication skills. How to apply the game principles, game-based learning elements, and competitive spirits in game-based language learning to carry out scientific and reasonable instructive teaching contents design for college students, how to arrange proper course, and how to set scenes of games appropriately and make a role-play, as well as the evaluation of teaching effects, above all are core issues that require further research. This research is only a preliminary attempt to explore the game-based learning in a Chinese university. The research sample is limited to 66 freshmen and the empirical cycle is limited to the teaching practice of these two courses. How to research on game-based language learning on a greater scale and depth, and study in a longer learning period is our next phase of work. Additionally, the game software “Digital Game-Virtual College” adopted in this study, though widely used by English learners in China, the functions of which are not rich enough, and existing limitations if more sophisticated learning tasks are given. Though we have carried out questionnaires on the design of game-based learning software and understood the college students’ needs and concerns on the game itself when learning spoken English, is also necessary to study how to use more improved game software to carry out more practice on language learning targeted. Furthermore, it is crucial to seek cooperation with game-based second language learning software companies, learn from successful game software cases in other fields about their design and implementation experience, and build a game-based language learning model with higher feasibility, better teaching effect, and more popular with students. With the more contextualized and intelligent development of language learning game software, it can help students carry out game-based oral language learning better, and improve students’ oral communication ability.

## Data availability statement

The original contributions presented in the study are included in the article/Supplementary material, further inquiries can be directed to the corresponding author.

## Author contributions

XX contributed to the acquisition and analysis of data, the design of the study, and the drafting of the manuscript. All authors contributed to the article and approved the submitted version.

## Funding

This work was supported by foundation of Guangzhou Jiaotong University Majors Construction Project “Characteristic Teaching Reform Project Foreign Language Teaching under the CLIL Framework: Research on the construction of curriculum language integration model” (K520222042); foundation of Characteristic innovation projects of universities of Guangdong Provincial Department of Education (2022WTSCX094); and foundation of Guangzhou Higher Education Teaching Quality and Teaching Reform Project (2022JXTD019).

## Conflict of interest

The authors declare that the research was conducted in the absence of any commercial or financial relationships that could be construed as a potential conflict of interest.

## Publisher’s note

All claims expressed in this article are solely those of the authors and do not necessarily represent those of their affiliated organizations, or those of the publisher, the editors and the reviewers. Any product that may be evaluated in this article, or claim that may be made by its manufacturer, is not guaranteed or endorsed by the publisher.

## Supplementary material

The Supplementary material for this article can be found online at: https://www.frontiersin.org/articles/10.3389/fpsyg.2022.1109370/full#supplementary-material

## References

[ref1] Alt D. Raichel N. (2020). Enhancing perceived digital literacy skills and creative self-concept through gamified learning environments: insights from a longitudinal study. Int. J. Educ. Res. 101:101561. doi: 10.1016/j.ijer.2020.101561

[ref2] Ghani N. A. Teo P.-C. Ho T. C. F. Choo L. S. Kelana B. W. Y. Adam S.. (2022). Bibliometric analysis of global research trends on higher education internationalization using Scopus database: towards sustainability of higher education institutions. Sustainability 14:8810. doi: 10.3390/su14148810

[ref3] Ghazal S. Singh S. (2016). Game-based language learning: activities for ESL classes with limited access to technology. ELT Voices-Int. J. Teach. Eng. 6, 1–8.

[ref4] Gomes L. A. Brasileiro T. S. A. Caeiro S. S. F. S. (2022). Sustainability in higher education institutions in the Amazon region: a case study in a Federal Public University in Western Pará. Brazil. Sustain. 14:3155. doi: 10.3390/su14063155

[ref5] Homer B. D. Plass J. L. (2014). Level of interactivity and executive functions as predictors of learning in computer-based chemistry simulations. Comput. Hum. Behav. 36, 365–375. doi: 10.1016/j.chb.2014.03.041

[ref6] Huizenga J. Admiraal W. Akkerman S. Dam G. T. (2009). Mobile game-based learning in secondary education: engagement, motivation and learning in a mobile city game. J. Comput. Assist. Learn. 25, 332–344. doi: 10.1111/j.1365-2729.2009.00316.x

[ref7] Hwang G.-J. Chen P.-Y. (2022). Interweaving gaming and educational technologies: clustering and forecasting the trends of game-based learning research by bibliometric and visual analysis. Entertain. Comput. 40:100459. doi: 10.1016/j.entcom.2021.100459

[ref8] Kapp K. M. The Gamification of Learning and Instruction: Game-based Methods and Strategies for Training and Education, San Francisco, CA: Pfeiffer. (2012).

[ref9] Kiili K. Moeller K. Ninaus M. (2018). Evaluating the effectiveness of a game-based rational number training - in-game metrics as learning indicators. Comput. Educ. 120, 13–28. doi: 10.1016/j.compedu.2018.01.012

[ref10] Li X. Zhao X. Xu W. (. A.). Pu W. (2020). Measuring ease of use of mobile applications in e-commerce retailing from the perspective of consumer online shopping behaviour patterns. J. Retail. Consum. Serv. 55:102093. doi: 10.1016/j.jretconser.2020.102093

[ref11] Liu Z.-Y. Shaikh Z. A. Gazizova F. (2020). Using the concept of game-based learning in education. iJET 15, 53–64. doi: 10.3991/ijet.v15i14.14675

[ref12] Mccandliss B. D. (2010). Educational neuroscience: the early years. Proc. Natl. Acad. Sci. U. S. A. 107, 8049–8050. doi: 10.1073/pnas.1003431107, PMID: 20421482 PMC2889531

[ref13] McLaren B. M. Richey J. E. Nguyen H. Hou X. (2021). How instructional context can impact learning with educational technology: lessons from a study with a digital learning game. Comput. Educ. 178:104366. doi: 10.1016/j.compedu.2021.104366

[ref14] McLoughlin C. Lee M. (2010). Personalised and self regulated learning in the web 2.0 era: international exemplars of innovative pedagogy using social software. Aust. J. Educ. Technol. 26, 28–43.

[ref15] Nalan A. (2015). Theoretical view to the approach of the edutainment. Proc. Soc. Behav. Sci. 186, 1232–1239. doi: 10.1016/j.sbspro.2015.04.081

[ref16] Ninaus M. Kiili K. McMullen J. Moeller K. (2017). Assessing fraction knowledge by a digital game. Comput. Hum. Behav. 70, 197–206. doi: 10.1016/j.chb.2017.01.004

[ref17] Oksana V. A. Elena V. Y. (2015). Edutainment as a modern Technology of Education. Proc. Soc. Behav. Sci. 166, 475–479. doi: 10.1016/j.sbspro.2014.12.558

[ref18] Osifo A. Improving collaboration in blended learning environments through differentiated activities and Mobileassisted-language learning tools. 15th International Conference Mobile Learning 2019, (2019). 3–10. doi: 10.33965/ML2019_201903L001

[ref19] Pásztor A. Magyar A. Pásztor-Kovács A. Rausch A. (2022). Online assessment and game-based development of inductive reasoning. J. Intelligence 10:59. doi: 10.3390/jintelligence10030059, PMID: 35997415 PMC9397073

[ref20] Perrotta C. Featherstone G. Aston H. Houghton E. Game-based Learning: Latest Evidence and Future Directions (NFER Research Programme: Innovation in Education). Slough: NFER. (2013).

[ref21] Plass J. L. Homer B. D. Kinzer C. K. (2015). Foundations of game-based learning. Educ. Psychol. 50, 258–283. doi: 10.1080/00461520.2015.1122533

[ref23] Réka Ö. Gyöngyvér M. (2022). Computer-based intervention closes learning gap in Maths accumulated in remote learning. J. Intelligence 10:58. doi: 10.3390/jintelligence10030058, PMID: 35997414 PMC9397034

[ref24] Schaaf R. (2012). Does digital game-based learning improve student time-on-task behavior and engagement in comparison to alternative instructional strategies? Can. J. Action Res. 13, 50–64. doi: 10.33524/CJAR.V13I1.30

[ref25] Schobel S. Saqr M. Janson A. (2021). Two decades of game concepts in digital learning environments - a bibliometric study and research agenda. Comput. Educ. 173:104296. doi: 10.1016/j.compedu.2021.104296

[ref26] Shaffer D. W. Halverson R. Squire K. R. Gee J. P. Video games and the future of learning (WCER working paper no. 2005-4). Madison: University of Wisconsin–Madison, Wisconsin Center for Education Research (NJ1) (2005). 87, 105–111, doi: 10.1177/003172170508700205

[ref27] Sun C. Shut V. Stewart A. E. B. Beck-White Q. Reinhardt C. R. Zhou G.. (2022). The relationship between collaborative problem solving behaviors and solution outcomes in a game-based learning environment. Comput. Hum. Behav. 128:107120. doi: 10.1016/j.chb.2021.107120

[ref28] Teng (2022). Incidental L2 vocabulary learning from viewing captioned videos: effects of learner-related factors. System 105:102736. doi: 10.1016/j.system.2022.102736

[ref29] Tomczyk L. Walker C. (2021). The emergency (crisis) e-learning as a challenge for teachers in Poland. Educ. Inf. Technol. 26, 6847–6877. doi: 10.1007/s10639-021-10539-7, PMID: 33897266 PMC8052202

[ref30] Wang B. Wu Z.-J. (2012). Considering of learning foreign language through edutainment-based on RPG game. Mod. Educ. Technol. 22, 77–79.

[ref31] White K. McCoy L. P. (2019). Effects of game-based learning on attitude and achievement in elementary mathematics. Netw. Online J. Teach. Res. 21:5. doi: 10.4148/2470-6353.1259

