# Supplementary material for: Subjective experiences and perceptions of learning a second language through digital games: A case study of Chinese college students
Source: Front Psychol. 2023 Jan 9;13:1109370. doi: 10.3389/fpsyg.2022.1109370 (PMC9868754; doi:10.3389/fpsyg.2022.1109370)
Supplement: Supplementary file 1 [file Data_Sheet_1.pdf]

Appendix A

A.1. Table 3. Questionnaire survey results of using game-based learning software "Digital Game-Virtual College" (I).

Aspects of questionnaire	No.	Questions	Control Group					Experimental Group				
			Not sure at all	Not very sure	average	Somewhat sure	Extremely sure	Not sure at all	Not very sure	Average	Somewhat sure	Extremely sure
Language Awareness	1	You often speak English in daily oral conversation consciously.	2	6	8	8	42	0	3	6	9	48
	2	You often use slang and idioms in daily oral conversation consciously.	3	7	10	21	25	1	6	9	22	28
Cognitive Awareness	3	You can freely introduce your personal information in social communication.	2	4	6	11	43	0	2	5	7	52
	4	You can introduce your hobbies to your friends clearly.	4	8	14	23	17	2	6	13	25	20
	5	You can introduce food spontaneously in dinner party.	6	11	15	28	6	4	7	16	26	13
	6	You can explain the scenery purposefully during your trip.	7	14	18	19	8	4	9	17	22	14
Social Awareness	7	You would take the initiative to try to be the boss of the company and deploy work to your subordinates.	10	16	24	9	7	6	11	20	17	12
	8	You will take the initiative to try to be a company employee and report to your superiors.	13	20	17	12	4	7	12	18	19	10
	9	You will take the initiative to try to be a customer and try to cut down the commodity price.	9	14	13	19	11	2	9	16	28	11
	10	You will actively try to be a seller and pursue profit maximization.	11	17	18	15	5	3	8	19	24	12
Emotional Awareness	11	You are willing to play the leading role in movies and TV dramas and perform love stories.	10	15	16	21	4	4	6	17	26	13
	12	You are willing to have emotional communication with your partner and cure him / her.	12	18	19	14	3	4	7	22	24	9

A.2. Table 4. Questionnaire results of game-based learning by using "Digital Game-Virtual College" software (II).

Timing of Survey	No.	Questions	Answer YES	Answer NO
Before Game-based learning	1	Do you like video games?	62	4
	2	Do you like using video games to aid your study?	49	17
	3	Have you ever been taught by the teaching method of game-based learning?	39	27
	4	Compared with traditional teaching, are you willing to accept the game-based teaching?	61	5
After game-based learning	1	Do you like video games?	65	1
	2	Do you like using video games to aid your study?	62	4
	3	Have you ever been taught by the teaching method of game-based learning?	66	0
	4	Compared with traditional teaching, are you willing to accept the game-based teaching?	65	1
	5	Do you often reduce your original study time due to playing games?	54	12
	6	Do you think game teaching can improve the relationship between teachers and students?	65	1
	7	Do you think game teaching would affect the teaching progress and adversely affect the classroom teaching?	55	11
	8	Do you think game teaching can reduce the difficulty of learning?	47	19
	9	Do you think game teaching is of great help?	62	4
	10	Times are changing. In the face of "information natives", game-based teaching is one of the main ways to move towards future education. Do you agree with this view?	62	4
	11	Do you find it challenging and fulfilling to design your learning content as a game for your peers?	61	5
	12	In the process of game learning, do you think rich and interesting game materials are very important?	64	2
	13	Through game learning, do you think it can stimulate your interests in learning?	64	2
	14	Do you think learning in the game can exercise your Manual dexterity and expression ability?	63	3
	15	Through game learning, do you think you can cultivate the students' team spirit?	63	2
	16	There is a bottleneck in the process of the game. Are you willing to acquire the knowledge further?	63	3
	17	In the process of game teaching, do you think the teaching game is closely related to the teaching contents?	63	3
	18	In the process of game teaching, can you involve in a the classroom as a team player soon?	64	2
	19	In the process of game teaching, can you participate in the classroom and actively cooperate with the teacher's teaching?	65	1
	20	In the process of game teaching, do you have sufficient opportunities to practice the skills you need to master?	63	3
	21	Is the layout and atmosphere of game teaching conducive to your study?	64	2
	22	Can game based learning give you a comprehensive and detailed understanding of what you have learned?	63	3
	23	Compared with other teaching methods, can you master knowledge points more easily in game teaching?	62	4
	24	Compared with traditional teaching methods, can you easily apply the knowledge learned in the process of game teaching to practice?	63	3
	25	Compared with traditional teaching methods, can game teaching be easier to cause your reflection?	62	4

26	Compared with traditional course teaching, what changes do you think game-based learning can bring to you?	Solid theoretical knowledge	35	31
		Improvement of professional skills	37	29
		Change of learning attitude	50	16
		Flexible application of knowledge and skills in work	48	18
		Improvement of enthusiasm in learning	54	12
27	Compared with traditional mode of teaching, what qualities do you think game teaching can bring you?	Stage performance	40	26
		verbal dexterity	55	11
		manual dexterity	48	18
		leadership	31	35
		Self-confidence	49	17
28	After game-based learning, based on your current understanding of game-based learning, what is your idea on it ?	Not helpful to study	0	66
		The form is over content, which is easy to distract and inefficient	3	63
		Too much emphasis on the process of the game and insufficient mastery of practical knowledge	8	58
		The process is easy, but the overall structure of knowledge cannot be realized from the game playing.	14	52
		Learn through practice, one can acquire much knowledge and have a better understanding	55	11
		Very effective, one can apply what he/she has learned in class into practice	57	9
29	Do you think that the game teaching can achieve the desired effect better than the traditional teaching method?		64	2
30	Compared with traditional teaching, do you think the assessment and behavior performance in game teaching are more reasonable and scientific?		61	5
31	Looking back on the game based teaching activities you have participated in, are you impressed by the fun rather than the knowledge itself?		60	6
32	Looking back on the game teaching activities you have participated in, do you think some of the activities are not closely related to the content in classroom?		13	53
33	Looking back on the game teaching activities you have participated in, do you think some activities take too much time, so that you don't know what you have obtained in the end?		16	50
34	If more classroom learning is conducted by game learning, will you focus more on learning than entertainment?		63	3
35	Are you willing to accept game-based teaching in future course learning?		62	4

A.3. Table 5. Questionnaire results of game-based learning by using "Digital Game-Virtual College" software (III).

Evaluation on game-based-learning software design	No.	Questions		Answers				
				Not important at all	Not very important	Fair	Important	Very important
Evaluation index of game-based learning software	1	When designing game teaching software, which of the following indicators do you think is the most important?	Entertaining nature	1	0	5	25	35
			Educative nature	1	0	4	18	43
			Rewarding	1	3	23	20	19
			Participation	1	0	5	26	34
			Artistry	0	1	23	20	22
			Competitiveness	0	3	15	25	23
			Teamwork	1	0	8	20	37
			Experience design	0	0	6	25	35
	2	Among the interesting indicators, which of the following indicators do you think is the most important?	Plot design	0	0	12	28	26
			Screen design (pictures, sound)	0	2	16	22	26
			Application of technologies such as helmet	1	6	19	18	22
	3	Among the educational indicators, which of the following indicators do you think is the most important?	Knowledge element	0	0	3	21	42
			Skill elements	0	1	3	25	37
			Emotional elements	0	0	7	29	30
			Value elements (ideological and political elements)	0	0	6	22	38
	4	Among the participatory indicators, which of the following indicators do you think is the most important?	Organization	0	0	5	28	33
			Degree of completion	0	0	12	26	28
			Abundant resources	0	1	10	23	32
Types of game-based software	5	From the close combination with the curriculum, the current game types are divided into knowledge embedded games and teaching support games. Which of the following types of games do you think is the most important?	Knowledge embedded games	0	0	8	32	26
			Teaching and learning supported games	0	0	12	28	26

Types of game-based learning software	6	Among the game types in the knowledge-embedded games based online course, which of the following do you think is the most important?	Difficulty	0	1	23	23	19
			Quantity	0	2	23	23	18
			Ways to play	0	1	12	28	25
			Interactivity	0	1	7	25	33
			Entertaining	0	0	7	26	33
	7	Among the game types in the teaching-supported games based online course, which of the following do you think is the most important?	Difficulty	0	1	22	25	18
			Quantity	0	2	22	24	18
			Ways to play	0	1	13	28	24
			Interactivity	0	0	9	27	30
			Entertaining	0	0	9	27	30
Design of reward mechanism for game-based learning	8	In the reward mechanism design of game learning, which of the following reward mechanisms do you think is the most important?	Regional ranking mechanism (class, school, region, national)	3	7	20	19	17
			Title / Title Mechanism	2	4	19	21	20
			Integral mechanism	2	3	16	26	19
			External means reward mechanism	1	1	19	24	21
			Virtual reward mechanism	2	6	15	22	21
